# Morbidité et mortalité hospitalière des maladies cardiovasculaires en milieu tropical: exemple d'un centre hospitalier à Lomé (Togo)

**DOI:** 10.11604/pamj.2014.17.62.2237

**Published:** 2014-01-26

**Authors:** Findibe Damorou, Soodougoua Baragou, Machihuede Pio, Yaovi M Afassinou, N'kenon W N'da, Soulemane Pessinaba, Tchaa Tchérou, Halé Attiogbé, Koffi Ehlan, Edem Goeh-Akue, Komlavi Yayehd

**Affiliations:** 1 Centre hospitalier universitaire Campus de Lomé, Service de cardiologie; 2 Programme National de Lutte contre les maladies cardiovasculaires; 3 Centre hospitalier Sylvanus Olympio de Lomé, Service de cardiologie

**Keywords:** Maladies cardiovasculaires, morbidité, mortalité, létalité, Afrique tropicale, cardiovascular diseases, morbidity, mortality, lethality, tropical Africa

## Abstract

**Introduction:**

Les maladies cardiovasculaires (MCV) occupent une place importante dans la mortalité en Afrique, situation inquiétante traduisant une transition épidémiologique rapide. La connaissance des groupes nosologiques les plus mortelles devrait aider à l’élaboration de politiques en matière de traitement et de prévention.

**Méthodes:**

Il's agit d une étude rétrospective du 1^er^ janvier 2006 au 31 décembre 2010, ayant inclus les patients hospitalisés pour une MCV selon la classification Internationale des maladies (CIM-10).

**Résultats:**

La fréquentation hospitalière pour maladie MCV a augmenté au fil du temps; les maladies hypertensives étaient le premier motif d hospitalisation (66.8%). Les autres pathologies fréquentes étaient la maladie thromboembolique (9.1%), les syndromes coronaires aigus (SCA) (7.3%), l'insuffisance cardiaque (5.5%), les cardiomyopathies (5.3%). La majorité des sujets étaient jeunes (âge moyen: 55.1 ans) et il n'y avait pas de corrélation entre la durée d'hospitalisation et l’âge: r = + 0.024, p = 0.09. La mortalité globale était élevée (11%) et les taux de létalité élevés dans le SCA (27.9%), l'embolie pulmonaire (25%) et la péricardite (25%).

**Conclusion:**

Les MCV sont responsables d'une hospitalisation croissante dans nos hôpitaux. L'absence d unité de soins intensifs cardiologiques et des méthodes de revascularisation coronaire, le manque de personnels qualifiés associés l'inexistence de sécurité sociale expliquent une mortalité élevée dans nos pays en voie de développement qui sans avoir achevé leur transition démographique entre en pleine transition épidémiologique.

## Introduction

Selon le Braunwald Heart Diseases, les maladies cardiovasculaires (MCV) sont devenues la plus grande cause de décès dans le monde dans la dernière décennie [1]. En 2008, l'Organisation Mondiale de la Santé (OMS) estimait à 17.3 millions le nombre de décès lié aux MCV dans le monde et ce chiffre devrait atteindre 23.6 millions en 2030 [2]. Entre 1990 et 2001, de tous les décès survenus dans les pays à revenus faible et intermédiaire, les décès liés aux MCV ont augmenté de 26% à 28%, traduction d'une transition épidémiologique rapide [3]. Tandis que les populations européennes et nord-américaines ont connues des mutations similaires en matière de démographie et de maladies pendant quelques siècles, les pays africains traversent des changements similaires juste en quelques décennies [4]. L'OMS estime qu'en 2020 la morbidité cardiovasculaire à laquelle devront faire face les pays africains aura doublée; les répercussions socioéconomiques auront probablement un impact négatif sur le développement de ces pays les rendant par conséquent plus pauvres [5].

Au Togo, les données de la littérature sur le sujet sont rares. Le but de cette étude était de déterminer la morbidité et la mortalité hospitalière des MCV dans le service de cardiologie du centre hospitalier universitaire (CHU) campus.

## Méthodes

Cette étude a été réalisée dans le service de cardiologie du CHU campus de Lomé, premier centre de référence en matière de cardiologie du pays.

### Population de l’étude

Il s agit d'une étude rétrospective descriptive, menée sur 5 ans, de janvier 2006 à décembre 2010, ayant inclus les dossiers de patients hospitalisés chez qui le diagnostic d'une maladie cardiovasculaire a été posé. Les dossiers incomplets ne comportant pas les éléments nécessaires permettant de retenir un diagnostic précis ont été exclus. Ainsi, sur les 962 dossiers de patients admis pendant la période d’étude, 118 ont été exclus pour données insuffisantes permettant de retenir le diagnostic d'une MCV, 844 dossiers de patients présentant une MCV ont alors été retenus.

### Définition des maladies cardiovasculaires

La définition des maladies cardiovasculaires a été faite selon la classification internationale des maladies (CIM-10), chapitre 09: maladies de l'appareil circulatoire [6]. Selon cette classification, l'hypertension artérielle (HTA) est considérée comme une maladie. Les comorbidités ont été définies par l'association d'autre affection cardiovasculaire à la cardiopathie de base. L'HTA a été parfois considérée comme un facteur de risque lorsqu elle est associée à une cardiopathie bien individualisée par la CMI-10.

### Les paramètres de l’étude

Les paramètres d’étude étaient les suivants: les données sociodémographiques (âge et sexe), les facteurs de risque cardiovasculaire (HTA lorsqu elle n est pas l'affection principale, diabète, tabac, obésité, dyslipidémie, hyperuricémie), le diagnostic retenu (fréquence, comorbidités), l’évolution de la maladie (durée d hospitalisation, mortalité, létalité).

### Définitions de variables et mesures utilisées

La surcharge pondérale a été classée après calcul de l'indice de masse corporelle (IMC) ou indice de Quetelet: IMC = poids/Taille^2^ (poids en kg; taille en mètre). Le surpoids a été considéré pour un IMC compris entre 25 et 29.9 kg/m^2^, et l'obésité pour un IMC 30 kg/m^2^ [7]. L'HTA a été définie par une pression artérielle supérieure ou égale 140/90 mm Hg et/ou un antécédent connu d'hypertension artérielle chez un patient sous traitement antihypertenseur [8]. Le tabagisme a été défini comme le fait de fumer au moins une cigarette par jour.

### Mesures des paramètres biochimiques

La cholestérolémie totale, la triglycéridémie, le taux de HDL-cholestérol, l'uricémie et la glycémie ont été déterminées à jeun par des méthodes enzymatiques pendant l'hospitalisation. La dyslipidémie a été définie pour une cholestérolémie totale > 200 mg/dl, et/ou un taux de LDL-cholestérol > 130 mg/dl, et/ou une triglycéridémie > 150 mg/dl, et/ou un taux de HDL-cholestérol < 40 mg/dl. Le Diabète a été définie pour une glycémie à jeun > 126 mg/dl à deux reprises, l'hyperuricémie pour une uricémie > 60 mg/l.

### Analyse des données

Les paramètres quantitatifs sont présentés sous forme de moyenne ± écart-type et les paramètres qualitatifs par l'effectif suivi du pourcentage. Le test de Khi deux a été utilisé pour les comparaisons de variables catégorielles et le test ANOVA a été utilisé pour les variables quantitatifs avec un seuil de significativité de p inférieur à 0.05. Le coefficient de corrélation de Pearson a été calculé pour établir la relation entre l’âge et la durée d hospitalisation. L'analyse des données a été effectuée à l′aide du logiciel Epi Info version 3.5.3.

## Résultats

### Caractéristiques sociodémographiques

**Figure 1:**
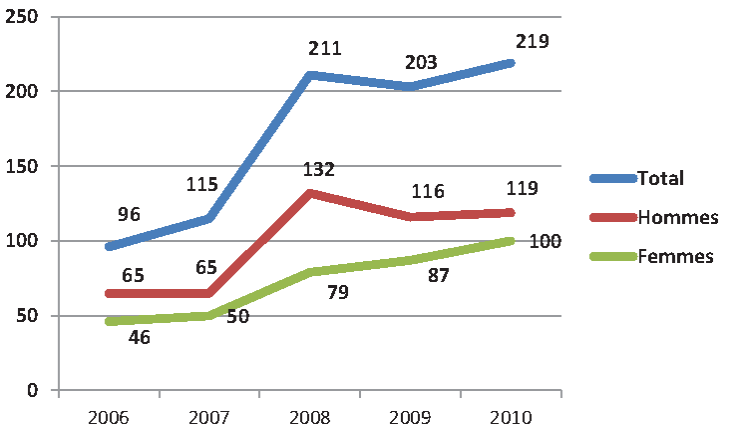
Evolution des hospitalisations dans le temps

Sur les 844 patients, il y avait 366 (43.4%) hommes et 478 (56.6%) femmes, soit un sex-ratio (H/F) de 0,76. La fréquence des hospitalisations évoluait de façon croissante allant de 96 cas en 2006 à 219 cas en 2010, cette tendance évolutive s observait aussi bien chez les hommes que chez les femmes (Figure 1). L’âge moyen des patients était de 55.1 ± 15.7 ans (extrêmes: 18-95 ans). Il était de 55.0 ± 15.4 ans chez les hommes contre 55.2 ± 15.8 ans chez les femmes, p = 0.87. Les patients âgés de 40 à 69 ans étaient les plus représentés avec 61.9% des hospitalisations (Table 1). Les FDR cardiovasculaire étaient retrouvés chez 89,3% des patients. Deux cent soixante-cinq malades (35.l1%) avaient un seul FDR cardiovasculaire.

**Table 1:** Répartition des patients par tranche d’âge et par sexe

	Total (n = 844)	Hommes (n = 366)	Femmes (n = 478)	P
**< 20 ans**	4 (0.5%)	2 (0.5%)	2 (0.4%)	0.81
**20-29 ans**	48 (5.7%)	12 (3.3%)	36 (7.5%)	0.0082
**30-39ans**	87 (10.3%)	45 (12.3%)	42 (8.8%)	0.09
**40-49ans**	173 (20.5%)	85 (23.2%)	88 (18.4%)	0.08
**50-59ans**	186 (22%)	80 (21.8%)	106 (22.2%)	0.91
**60-69ans**	164 (19.4%)	65 (17.7%)	99 (20.7%)	0.28
**70-79ans**	125 (14.8%)	56 (15.3%)	69 (14.4%)	0.72
**≥80ans**	57 (6.7%)	21 (5.7%)	36 (7.5%)	0.30

Les FDR cardiovasculaire étaient présents chez 318 hommes (86.9%) contre 436 femmes (91.2%), p = 0.04. Une prédominance féminine a été retrouvée pour l'obésité (p = 0.014) alors qu il y avait une prédominance masculine pour le tabac et l'hyperuricémie (p < 0.001) (Table 2). Parmi les patients obèses, 32 (28.6%) avaient une surcharge pondérale, 46 (41.1%) avaient une obésité modérée (IMC: 30 à 35 Kg/m^2^), 23 (20.5%) une obésité sévère (IMC: 35 à 40 Kg/m^2^) et 11 (9.8%) une obésité morbide (IMC > 40 Kg/m^2^).

**Table 2:** Répartition des FDR cardiovasculaire en fonction du sexe

	Hommes (%)	Femmes (%)	P
**HTA**	266(72.67)	379(79.28)	0.2
**Diabète**	60(16.4)	79(16.5)	0.95
**Tabac**	17(4.6)	3(0.6)	< 0.001
**Obésité**	38(16.6)	74(25.4)	0.014
**Dyslipidémie**	156(77.6)	241(84.3)	0.06
**Hyperuricémie**	136(76.4)	130(57.5)	< 0.001

### La morbidité

La maladie hypertensive était le premier motif d hospitalisation avec 66.8% des cas. On notait 4.9% de cas d'insuffisance cardiaque dont l’étiologie n a pu être élucidée. Les maladies les moins fréquentes (6.6%) étaient les myocardites, les endocardites, l'artériopathie oblitérante des membres inférieurs (AOMI), l'insuffisance veineuse, les valvulopathies, le coeur pulmonaire chronique et les cardiopathies congénitales de l'adulte. La prédominance féminine retrouvée de façon globale ne se confirmait pas lorsque les MCV étaient prises séparément (Table 3).

**Table 3:** Répartition des MCV selon le sexe

	Total (n = 844)	Homme (n = 366)	Femme (n = 478)	Sex ratio (H/F)	P
Maladie hypertensive	564	234 (63.9)	330 (69)	0.7	0.11
Insuffisance cardiaque non élucidée	41	21 (5.8)	20 (4.2)	1.05	0.29
Cardiomyopathie	45	20 (5.5)	25 (5.2)	0.8	0.88
Maladie thromboembolique	77	35 (9.56)	42 (8.76)	0,83	0,69
SCA	61	32 (8.74)	29 (6.06)	1.1	0.13
Péricardite	20	10 (2.7)	10 (2.1)	1	0.54
Cœur pulmonaire chronique	5	2 (0.5)	3 (0.6)	0.6	0.87
Valvulopathie	12	5 (1.36)	7 (1.46)	0.7	0.9
Myocardite	6	1 (0.3)	5 (1.0)	0.2	0.18
Endocardite infectieuse	5	2 (0.5)	3 (0.6)	0.6	0.87
AOMI	5	3 (0.8)	2 (0.4)	1.5	0.45
CC	2	2 (0.5)	0	-	-
Insuffisance veineuse	1	0	1 (0.2)	-	-

SCA= Syndrome coronarien aigu, AOMI= Artériopathie des membres inférieurs, CC= Cardiopathie Congénitale. Les données sont en n (%).

L'insuffisance cardiaque toutes étiologies comprises était retrouvée chez 463 patients (54.8%). Les étiologies étaient l'HTA dans 74.7% (346 cas), les cardiomyopathies dans 9.7% (45cas), l'IDM dans 3.0% (14 cas), les valvulopathies dans 2.6% (12 cas) et le coeur pulmonaire chronique dans 1.1% (5cas). L’étiologie n a pas été retrouvée dans 8.8% des cas. Les patients porteurs de cardiopathies rhumatismales (29 ± 12.8 ans) et de cardiopathies congénitales de l'adulte (30.5 ± 16.3 ans) étaient les plus jeunes (Table 4).

**Table 4:** L’âge moyen des patients selon le type de MCV

Type de MCV	Age moyen (années)	Extrêmes (années)
Maladies hypertensives	57.0 ± 14.7	18-95
Insuffisance cardiaque non élucidée	50.9 ± 18.6	23-92
Cardiomyopathie	46,2 ± 16.5	23-87
Embolie pulmonaire	53.1 ± 16.6	22-87
Thrombophlébite	52.1 ± 16.5	26-92
Angor instable	54.0 ± 16.7	22-92
Infarctus du myocarde	63.6 ± 9.1	42-84
Péricardite	46.7 ± 14.1	23-75
Cœur pulmonaire chronique	49,3 ± 11,1	32-69
Valvulopahies non rhumatismales	44.8 ± 17.5	21-68
Myocardite	57 ± 14.84	35-75
Endocardite	46 ± 22.84	18-77
Artériopathie oblitérante des membres inférieurs	53.6 ± 16.3	9-67
Cardiopathies rhumatismales	29 ± 12.8	18-43
Cardiopathies Congénitales à l’âge adulte	30.5 ± 16.3	19-42

MCV: maladies cardiovasculaires

### La durée d hospitalisation

La durée d'hospitalisation moyenne était de 8.89 ± 6.45 jours avec des extrêmes de 1 et 61 jours. Elle était de 9.37 ± 6.25 jours (extrêmes: 1-52 jours) chez les hommes et de 8.52 ± 6.58 jours (extrêmes: 1-61 jours) chez les femmes, p = 0.06. Il n'y avait pas de corrélation significative entre la durée d hospitalisation et l’âge: r = + 0.024, SE = 0.014 et p = 0.09. Le Table 5 montre la durée d hospitalisation moyenne en fonction des tranches d âge.

**Table 5:** Durée d'hospitalisation par tranche d’âge

Durée d'hospitalisation	Durée (jours)
**Selon la tranche d’âge**	
<20 ans	9.75±4.78
20-29 ans	7.75 ± 5.15
30-39 ans	8.83 ± 5.67
40-49 ans	8.37 ± 5.80
50-59 ans	8.38 ± 6.70
60-69 ans	10.04 ± 7.96
70-79 ans	9.47 ± 6.03
≥80 ans	8.35 ± 5.37
**Selon le type d'affection**	
Maladie hypertensive	8.29 ± 6.01
Insuffisance cardiaque non étiquetée	8.28 ± 5.09
Cardiomyopathie	8.84 ± 7.84
Maladie thromboembolique	11.06 ± 6.11
Syndrome coronarien aigu	10.88 ± 9.76
Péricardite	11.8 ± 4.2
Les MCV les moins fréquentes	10.87 ± 6.61

MCV moins fréquentes = valvulopathies, endocardite, myocardite, cœur pulmonaire chronique, artériopathie oblitérante des membres inférieurs, Cardiopathie Congénitale, insuffisance veineuse.

### Les comorbidités

Les comorbidités cardiovasculaires étaient retrouvées chez 91 patients dont 38 hommes (10.4%) et 53 femmes (11.%), p = 0.74. L âge moyen était de 60.8 ± 13.4 ans (extrêmes: 22-92 ans). Elles comprenaient les troubles du rythme supraventriculaires dans 49 cas (58.2%), le bloc auriculo-ventriculaire de haut degré dans 6 cas (6.6%). Les comorbidités non cardiovasculaires étaient représentées par l'accès palustre chez 159 patients (18.8%), les bronchopneumopathies dans 58 cas (6.9%), l'insuffisance rénale terminale dans 46 cas (5.4%), les cancers dans 9 cas (1.1%).

### La mortalité

**Figure 2:**
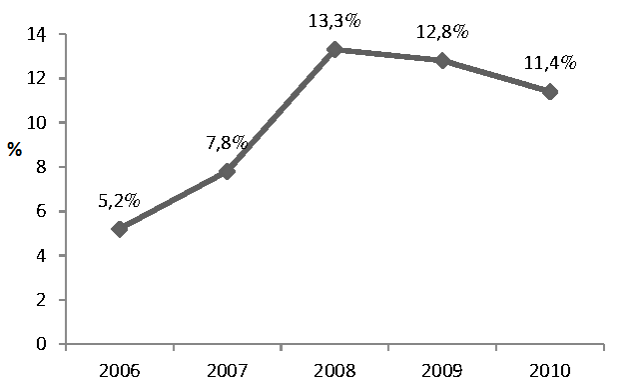
L’évolution du taux de mortalité dans le temps

**Figure 3:**
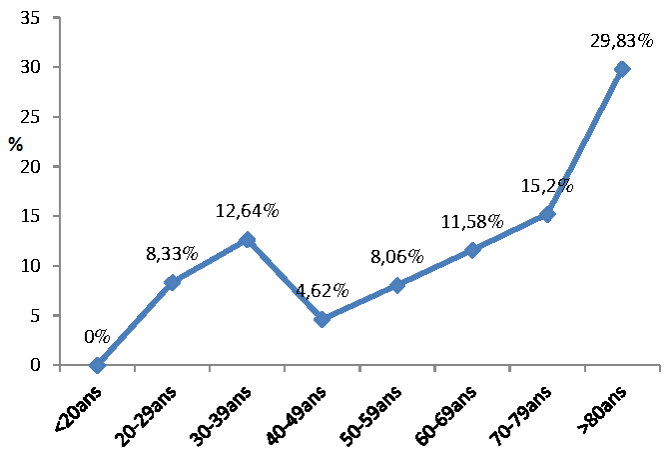
Taux de mortalité en fonction des tranches d’âge

Nous avons dénombré 93 décès soit un taux de mortalité global de 11%. Ces décès étaient composés de 40 (10.9%) hommes et 53 (11.1%) femmes, p = 0.94. Entre 2006 et 2010, le taux de mortalité était passé de 5.2% à 11.4%. La Figure 2 montre l évolution de la mortalité dans le temps. L’âge moyen des patients décédés était de 61.5 ans ± 17.0 ans (extrêmes: 28 et 92 ans). La répartition des décès selon les classes d âge montre un taux plus élevée chez les patients de plus de 70 ans (Figure 3).

La mortalité spécifique était plus importante dans la maladie hypertensive (5.9%) et les SCA (2.0%) qui constituaient les premières causes de décès. Les taux de létalité étaient plus élevés dans le SCA (27.9%), l'embolie pulmonaire (25%) et la péricardite (25%) (Table 6). La durée d'hospitalisation moyenne des patients décédés était de 8.7 ± 8.9 jours (extrêmes: 1 à 58 jours) contre 8.9 ± 6.1 jours chez les patients non décédés, p = 0.76.

**Table 6:** Mortalité spécifique et létalité des MCV

	Effectif des hospitalisés	Décès	Mortalité spécificité (%)	Taux de létalité (%)
Maladies hypertensives	564	50	5.9	8.9
Insuffisance cardiaque non élucidée	41	5	0.6	12.2
Cardiomyopathie	45	5	0.6	11.1
Embolie pulmonaire	28	7	0.9	25
Syndrome coronarien aigu	61	17	2.0	27.9
Cœur pulmonaire chronique	5	1	0.1	20
Péricardite	20	5	0.6	25
Endocardite	5	1	0.1	16.7
Myocardite	6	1	0.1	20
Valvulopathies	12	1	0.1	8.3

## Discussion

Entre 2006 et 2010, on a observé une augmentation de la fréquence des hospitalisations avec comme principaux groupes nosologiques les maladies hypertensives, la maladie thromboembolique veineuse, les SCA et les cardiomyopathies. La majorité des patients étaient d’âge jeune avec une importante proportion de femmes. Le taux de mortalité était de élevé et les affections les plus létales étaient les SCA.

Le Togo fait partie des pays qui sont aux premiers stades de la transition épidémiologique avec un taux de mortalité aussi bien élevé pour les maladies transmissibles que pour les maladies non transmissibles [9, 10]. L'augmentation de la fréquence des hospitalisations pour MCV pourrait traduire une augmentation de l'incidence des MCV dans nos populations. L'urbanisation a non seulement fait apparaitre le concept de transition démographique mais aussi celui de transition épidémiologique directement liée aux changements comportementaux qu'elle a entrainés; ainsi, l'accroissement de la prévalence des FDR cardiovasculaire dans la population — tabagisme, obésité, inactivité physique — provoqué par le changement du mode de vie dans nos pays serait la principale cause de cette recrudescence des MCV [9].

Les groupes nosologiques prédominants étaient les maladies hypertensives, la maladie thromboembolique veineuse, les SCA et les cardiomyopathies. Contrairement aux données des années 1990-2000 où les l'essentiel des MCV étaient composées de maladie hypertensive et de valvulopathies notamment rhumatismales [11, 12], on note une augmentation progressive de la fréquence des SCA en Afrique comme le montre l’étude de Suliman à Khartoum en 2011 [13]. Même si ces résultats contrastent fortement avec ceux retrouvés dans les pays développés où les coronaropathies constituent la majorité des hospitalisations, la part des coronaropathies dans la morbidité cardiovasculaire en Afrique va croissant et le manque de moyens de revascularisation alourdit le pronostic de cette affection faisant d'elle l une des affections les plus létales.

Plus de la moitié des patients étaient des femmes. Des études africaines plus anciennes notait une prédominance masculine allant de 54 à 64% des patients selon les séries [11, 14, 15]. La prédominance féminine pourrait être liée à la prédominance féminine significative retrouvée dans la distribution des FDR cardiovasculaire (p = 0.04) notamment la forte prévalence féminine de l obésité (p = 0.014) et l'espérance de vie plus élevée chez les femmes [9, 16]. De même, il existe de nos jours chez les femmes africaines, une expansion d'un certain nombre de FDR à la faveur du développement que sont l'utilisation des contraceptifs oraux et l'hormonothérapie de remplacement post-ménopausique [17, 18]. La majorité des patients étaient jeunes (âge moyen: 55.1 ans et tranche d âge la plus représentée était celle comprise entre 40 et 69 ans) comme il est le cas dans d autres études africaines: 52 ans au Nigeria [19]; Ce fait contraste très fortement avec ce qui est constaté dans les pays développés où on note une forte proportion des sujets âgés (âge moyen = 67 ans dans les unités de soins intensifs cardiologiques (USIC) d Iles de France [20]).

Les FDR cardiovasculaire étaient retrouvés chez 89,3% des patients. Une étude faite en Malaisie sur 3772 patients a révélé que 87% patients présentaient au moins un FDR cardiovasculaire [21].

La durée d'hospitalisation était en moyenne de 8,9 jours ± 6,4 jours, comparable à celle rapportée dans d'autres études africaines (9 ± 7 jours au Nigeria en 2008) [19]. La durée d'hospitalisation des patients vivants n’était pas statistiquement différente de celle des patients décédés. Par contre Ansa [19] avait trouvé une différence significative (p < 0.001) avec une durée plus courte (7 jours) chez les patients décédés.

Le taux de mortalité était élevé avec une létalité très importante dans les SCA. Diallo [11] au Mali rapportait une mortalité de 12.6% en 1994 et Ansa [19] au Nigeria 12.4% en 2008. En effet, les taux de mortalité cardiovasculaire restent élevés dans nos hôpitaux du fait de l'absence d unité de soins intensifs cardiologiques, la non disponibilité des méthodes de revascularisation coronaire, l'insuffisance de personnels qualifiés. Tous ces facteurs sont aggravés par l'absence de sécurité sociale. Ce sont en fait des problèmes que nos pays en voie de développement connaissent depuis le 20^e^ siècle et qui persistent en ce début de 21^e^ siècle du fait de l'instabilité socio-politique et économique qui perdure dans ces états. La mortalité cardiovasculaire a par contre régressé dans la majorité des pays industrialisés [22]. En Europe, une étude faite dans 27 pays avait noté une diminution de la mortalité liée aux maladies coronaires de -33% chez les hommes et de -27% chez les femmes entre 1985-1989 et 2000-2004 [23].

L’âge moyen des patients décédés était de 61,5 ans dans cette étude alors qu'il était d'environ 80 ans dans les USIC d’îles de France en 2004 [20]. L'OMS estimait que 46.7% des décès liés aux MCV dans les pays en voie de développement en 1990 survenaient avant l’âge de 70 ans [24]. La persistance de ce phénomène deux décennies plus tard démontre l’échec des politiques de santé qui ont jusqu'ici été menées dans ces pays.

### Limites de l’étude

Le diagnostic de certaines maladies n'a pu être réalisé du fait du sous équipement de nos structures sanitaires (dissection aortique, anévrisme aortique). Les évasions des hôpitaux, et les transferts sans feed-back de certains patients ont pu modifier l'appréciation de l’évolution et de la durée du séjour de certaines pathologies.

## Conclusion

Les MCV sont responsables d'une hospitalisation croissante dans nos hôpitaux. La mortalité était élevée et la létalité importante due aux difficultés dans la prise en charge des SCA et de l'embolie pulmonaire. L’équipement des structures sanitaires, basée sur la promotion des techniques de revascularisation, associé à une sensibilisation de la population sur les MCV et les facteurs de risque devraient aider à réduire la morbidité et la mortalité des MCV dans notre milieu.
